# A New Era in Dental Practice

**Published:** 1895-02

**Authors:** 

**Affiliations:** Philadelphia.


THE
AMERICAN JOURNAL
—OF—
DENTAL SCIENCE.

VOL. XXVIII. BALTIMORE, FEBRUARY, 1895. NO. 10.

ARTICLE I.

### A NEW ERA IN DENTAL PRACTICE.

BY PROF. FLAGG, PHILADELPHIA.

Through your courtesy* I am with you again, to reveal the silence of years which has culminated in what I am pleased to call "A New Era in Dentistry." Forty-five minutes is but a moment, which you accord me to condense forty years of active labor and practice. I can not blame you for the restriction, as I have always written exhaustively and hours have been consumed by me, leaving no chance for discussion at the same meeting. I will hold to your order, and leave for the future other chances for detatls and the consummation of the work, that my efforts may not have been in vain.

The great Master once said, "Neither will they be persuaded, though one rose from the dead," The profession of dentistry, as that of medicine, is demanding, "What shall we do to be saved?" We are in the light of

*New York Odontological Society.

the waning days of the nineteenth century, asking each other for some system by which we can be saved the humiliation of failure in dental practice to save the human teeth, one upon which each and all can rely.

Before we can attain to any true system that shall work universally, as in the laws of interchangeable mechanics, we must accord to some one the right from a life of precedents to be umpire in his line of work.

The title of this paper is an assumption, and all I ask is to follow, and for once get out of the old ruts and walk upon the broad plane of liberality and common sense, and with unselfish eye and charity calmly act and show that something good can come out of Nazareth.

Every journal bears upon its pages the desire of men to flee to a practice that will bring universal good results. Let us ask, where are our failures? Are they in men or things or materials? Are they in false education? Is it that new difficulties arise to baffle every past partially-successful effort? Have we really been successful in anything we have done to insure us in following the same old cause? The cry is for new light and new methods. If the world knew how few teeth are saved by us for any number of years, they would not spend time, health and money, but give up to the juggernaut of destruction, and extraction would be the rule, even with the wealthier classes. The poor and medium classes have had to give up their teeth in early life because of failure upon failure of even the best men. Then it is a fact, dentistry of the present hour is not near the goal of perfection in practice we long for. Why is it? Is there any relief? When shall we commence in our declaration of failure?

### THE PRIME NECESSITY.

First. Men. With all the vaunted advantages of colleges everywhere, how few who enter their sacred precincts are fitted by nature, general education, special talent, surroundings, or by long family precedents! Could we but be as true to principles and bold to assert as was Plato when he had inserted over the garden-gate to his studio, "Let none enter here who know not geometry," we would then have some hope for the future.

Any one can be taught to fill a cavity with the instruments and material at our disposal, but when to do it and how it should be shaped to insure its future usefulness is quite another consideration. We must not teach men that our art is solely to allow teeth to decay and fill our coffers from Nature's weakness. Are you prepared to-night to lay down your life as the missionary, and for the good of humanity attempt to save a tooth in its purity whether fortune in gold favor you or not?

Are you ready to ignore self and be the benefactors of your race, and adopt a system that promises to save more teeth, more pulps, give greater beauty, and more usefulness? If not, then step aside.

Medical men can well ask for "preventive medicine," for every honest M. D. soon learns to give less and less medicine, and rely not only on Nature, but in knowing the laws of hygiene, even if he be dethroned for his empiricism.

We, as dentists, have an entirely different field, for we have millions coming to us where no law of prevention can be applied. We can only save and restore the lost structure by our cunning and art.

With the rising generation, we can do, if we have a system to prevent the ravages so universally found in the human race, that which will preserve a much greater number in their purity and also check decay without filling with gold and other materials, and drive from our practice artificial substitutes in the majority of cases.

This leads us to a most important matter—ignoring the "greed for gold." If we are ever so perfect in our manipulation and able to preserve, yet if we do not have that charity to purge ourselves from the "lust for gain," and do for others as we would do for our own families, it will not bring us success at the end of our journey.

### GENERAL CAUSES OF FAILURE.

Let us take up the general causes of failure. We cannot ignore the almost utter worthlessness of tooth-structure. This we will call the "predisposition" My experience is that we are more degenerate every year, and the fight is harder to save teeth. It requires wisdom, foresight and skill to dare attempt the anticipation of caries. I have attempted to make the effort. *True: anticipation, as a general rule, cannot be relied on* by the average dentist. But there is much that can be done to check caries in its very incipiency and that without filling.

The materials as substitutes for lost structure we will now take up. Gold is acknowledged by the profession generally to be preeminently the best, and we want no better testimony than to look into any and every mouth and behold gold, gold, gold. Fillings no larger than pin-points and heads dotting every valley. Men talk and write gold, and yet they deplore it does not save. They ask for the reason why? The "New Departure" said "*never use it.*" Here are two extremes, and neither has shown why either should be practiced. You ask for testimony. I refer you to the many kinds of gold and preparations or forms of gold, each manufacturer claiming special features for success; the immense quantity of it used; the cases that have been filled and refilled. I filled a superior central incisor last week with Abbey's gold that had been filled fifteen times before. There was left for me a tooth with living pulp with the palatal surface one-half gone and both mesial and distal surfaces involved to the cervix. Think of it! None of you can deny that gold is the idol of the profession, and a man who talks against it is risking his reputation.

Gold, *per se*, is good for preserving tooth-structure. Compatibility has nothing to do with it. Adaptability is all of it! If tooth-structure is worthy as a base and a man knows how to line the walls of the cavity, it will preserve, provided the cavity is rightly shaped and the contour is given such form as to preclude any possibility of the active causes of caries. There is no greater error made than to suppose it is necessary to have various qualities and forms of this metal.

### Idolize it.

Make to yourself an idol in this metal, and in spite of the manufacturers, follow it; learn to manipulate one kind, and you have all you can ask, and there will be less failures. But the sin is not only in seeking for some better form of gold, but in learning how to use but one or two, and have the conscience and honor to confess that the fault is in yourself, and discriminate when and where it should be used. They tell us it quite always fails at the cervix; and, to attest this, they reproach the gold and use amalgam or gutta-percha at this point, It is not the gold, but it lies in you and your judgment and conscience. Franklin showed he was a philosopher when he said he "would not give much for a mechanic who could not bore with a saw and saw with a gimlet."

Some dentists have piled up around them every new instrument that comes out,—every new plugger-point with serrations of greater or less depth and extreme fineness and all sizes,—every form of mallet from the thud blow to the most approved power mallet. And withal, they fail, but few recognizing the fact that perfectly smooth points will do the whole business perfectly in adapting one fold of gold to another though perfectly burnished.

Tin and gold and hand pressure is another proof of gold's failure when used alone. I could multiply proofs of failure, but enough,—gold is a failure!

### The Plastic Crutch.

Take amalgam. Look at the tons of it used; yet, so many men say they do not have it in their office.

Do you ask for proof of failure?

Take the craze for "copper amalgam." What a curse it has been to the profession and their patients! I never used it once! I saw what it was when I looked into patients mouths when I was abroad. Had they known what proper contouring was they would never have used it so universally in all cavities.

It has been a stench in the nostrils of nearly every American dentist.

Has amalgam any good qualities in itself? Have we any good alloys?

How many men know how to use it and get the best results? What curses have been heaped upon its hallowed head? Where is the man who dares say he uses it? Anathemas come thick and fast from the M. D.'s of both old and new school as if they were the Supreme Court to sit upon its merits.

Several years ago, at one of the first meetings of our Odontological Society of Pennsylvania, one of its oldest and most prominent members went so far as to place on record in the Proceedings,—"All men, falling away from manipulative ability, lean on plastics." To-day he is experimenting to find an ideal alloy to help him out in his failures with gold. How sad such a record! Who among you so mean as to deny using it and without "apologizing"?

I say it is one of the grandest filling materials we have,—and while I have spent nearly thirty years in inventing power mallets and special smooth oval points for gold work, I confess I throw all aside very quickly, and while I delight to wield the electric or mechanical mallet in distancing time and space with gold, yet I use more amalgam to-day than ever before in my life.

I am thankful I had the courage and principle to stand by it, and recommended and show how much could be done when condensed under Japanese bibulous paper. To me it is a sheet-anchor for the class of cases every day coming to me from others. If you use amalgam and wish to make your practice a success with it, learn how to manipulate it and prepare your cases for its adaptation.

### ANOTHER.

Oxyphosphate,—what of it?

Is it good as a permanent filling alongside of gold or amalgam? Is it a failure also?

What does the profession say of it?

Everywhere you hear the cry, it gives way at the cervix in all cases and soon wears away, and is not fit for contour or permanent work. It is not to be relied upon!

If this be true, then it is useless to place it in carious teeth.

That it will preserve tooth-structure from further decay admits of no doubt whatever.

That it will preserve the contour of the tooth is certain in the bulk of cases.

That it will not destroy the pulp in near contact with it is equally sure.

That it will preserve tooth-structure with nearly all the decay left in the cavity and without much or really any shaping is incontestable, which, further on, I will show for a fact that I am willing to stand by and from a part of the new era of which I am here to speak. It is beyond value when you know how to mix it, how to manipulate it, how to shape it; how to treat it before you remove the dam or allow it to get wet; how to treat the phosphoric acid, to keep it from crystallizing, insuring you thereby a better result in every way; and when proper precautions are taken with these fillings, how inestimable are the results, and beyond cavil and doubt!

I cannot say too much for it,—a good article. But, just here let me say that, when you can get a good article, use no other kind in the mouth; as I forgot to tell you of amalgam, as with gold, use one kind only, that will work as well under water as above the surface. The oxyphosphate, of course, will have to be kept absolutely dry to be a perfect success.

### AN IDEAL MATERIAL.

In nearly every sense I know of nothing so important as pink base-plate gutta-percha.

To teach you how I use it for the preservation of the human teeth, both temporary and permanent, will be the foundation of a system that, if I have had any success at all, I can attribute to this one article as much as or more than any other filling-material.

In conjunction with this I cannot overestimate the value of the discovery of the laws of articulation and the articulator that bears my name, and of which I am more proud than of all my other productions. Without knowing what I do of articulation, the gutta-percha might never have been seen in the same light by me.

Let us review, now, before I enter upon this simple revelation of truth, what and wherein are our failures.

### LIST OF FAILURES.

I told you, poor elements in tooth-structure as the grand predisposing cause of failure.

Worthlessness of tooth-structure as a very great cause of failure, let us be ever so competent and with the best of materials for restoration.

The materials as substitutes for lost structure,--gold, amalgam, oxyphosphate, gutta-percha and tin,—and how failure comes from each and all.

The failure that comes from not understanding the laws of articulation, and which shows how the loss of one tooth affects all the teeth of both jaws.

The failure that result from the indiscriminate cutting of approximal surfaces in filling, and the great changes of relationship between the upper and lower dentures.

And the failures that ensue from the want of a definite system in knowing what to do in the keeping of the original articulation, and, when lost from bad dentistry, restoring it again to its proper relations that each tooth will bear its exact burden and no undue pressure be brought to bear upon any one.

The importance, finally, of those laws and this system in the prevention of recurrent caries and the blotting out of the greatest cause for pyorrhœa alveolaris,—which comes largely from undue pressure and use of the teeth thrown out of arrangement by non-restoration of contour.

I cannot enumerate all the causes as now acknowledged by the profession. Look into every journal, go into every depot, talk with every dentist you meet, scan the proceedings of every society, go where you will, and we can infer from it all.

Dentistry is a failure, because it can neither anticipate nor prevent decay superficially or arrest it when substitutes are made to take the place of lost tooth-structure.

We have labored in vain, and I am invited here to tell you whether I am satisfied with the practice I have instituted after forty years of servitude.

To say that I am really satisfied is not true. But I am conscious that my practice shows that I not only anticipate successfully, but preserve teeth superficially decayed without filling, and, when too far gone for this practice, then the conscientious use of the materials we have at hand enables me to snatch from the ravages of the "tooth of time."

Yes, I am happy to tell you how much can be done to rescue our profession from its perilous practice. But, I know you will not adopt what I tell you! Or at least the bulk of dentists will not, for they will fear starvation.

### BRAINS A BIG ITEM.

If you can have the courage to charge a patient as much for tin, amalgam, oxyphosphate, gutta-percha or beeswax,—which is a valuable material to save trouble,—then you have accepted the highest creed I offer you in my "New Era." Unless you can dare to face the public and say to them brains must be paid for, and all our operations are upon brain work as the standard of price, then do not accept my creed,—go on as aforetime. Hobbs, the celebrated locksmith and maker of the first complicated bank-locks, which were marvels of ingenuity, was brought before a bank committee to have him show cause why he asked such high prices for his inventions. They had him take the lock to pieces, and inquired what each piece would cost to duplicate it. When the sum total was made they found it did not foot up the price Hobbs asked for the completed lock. How is this, Mr. Hobbs? He then looked over all the items, and said to them, "Gentlemen, there is one item you have left out." They could not tell what it was,—when, to their chagrin, he said, "Brains."

But with this blot upon my career, "high charges," I am proud; and, if any one thing has added to my success above all else, it has been in daring and boldness to make people pay what I believed my brain, as a machine, was worth.

I often say to my patients now, when speaking of prices and they want an estimate made, I cannot do it, for "I have no more right to cheat myself than to cheat you."

Then, I am sure you will agree with me that this first article of my creed is worthy of following. If not, then do not listen to my simple system of practice.

It is not as manual laborere we can show our highest skill! No: one piece of timely advice—the extraction of one tooth; the teaching how to use a brush and what kind to use; and in many ways, where no labor at all is performed—is worth hundreds of dollars, and besides, a deep gratitude for the salvation from vandalism and sacrifice of Nature's most beautiful pearls and God's grandest piece of architecture.

Let us all dare to do right, even if we get no immediate compensation further than our own approving conscience.

Let us all dare to stop when we are in doubt, and the kindly consultation with another brother practitioner may be of the greatest value to us and our patient.

If we must link ourselves at all to the medical men, let us emulate their example in one thing at least,—anticipate medicine, or, as they have it, "preventive medicine."

As I have previously told you in this article, I have held opinions for many years that with every disadvantage impeding our course we can anticipate and cheat the "tooth of time" of its ravages. Yet it is a dangerous remedy in the hands of ignorance.

### Must Have Sole Charge.

Unless we can have sole charge of patients from the second year on, and no one else to interfere, we cannot hope to do our fullest duty and found a practice for all men to follow.

Ignorance, stupidity and downright dishonesty give us more trouble than we like to admit.

I have told you only of a few of the causes of our failures. One, above all the rest, faces us, and a wail is sent up everywhere, "Recurrence of decay at the cervical border;" no man yet has dared to say he was conqueror.

The next most treacherous is what is known as pyorrhœa.

I never use gold in the temporary teeth, seldom amalgam or tin, save on grinding surfaces, where cavities are very small or very large and no pulp involved in the part, and oxyphosphate very seldom, and only where I can keep it perfectly dry. Not that any of these articles are not valuable, but the preparation of cavities and the situation of decay, the near approach to the pulp of nearly every proximal decay and the age of the subject preclude their use. Never demoralize any youthful client by much excavation or formidable show of instruments, or by slow, sluggish movements. My aim is expedition; as few minutes in the chair as possible; inflicting but little pain and inconvenience,—gentleness, kindness, and yet positiveness.

My greatest ally as the filling-material is pink gutta-percha, such as is used for base-plate, and further on you will see how far I use it in the treatment of the permanent teeth. Aside from its use for a stopping on all approximal surfaces, there is one grand object in view to be ever held in mind, the importance of the position of the first permanent molar when it emerges. Unless this base column, or abutment if you please, is not kept well back towards the ramus, then *irregularity will come to the incisiors.* It is not enough to merely stop decay and stuff in amalgam or oxyphosphate; we must keep the temporary molars from approaching each other more than normal, and prevent the alveolar processes from encroachment and absorption from direct pressure of the roots of the temporary molars, which is invariably the case when the approximating surfaces are cut by caries and allowed to trespass on each other. We cannot use a separator here to gain space; we dare not cut or shape the cavities for a metal filling for fear of the pulp. What is to be done?

If possible, as soon as the least decay is noticed on the approximal surfaces and you can get in from the crown or on the buccal sides with the least excavating, by hand or machine, if it must be used, whether you can keep the cavity dry or allow it to remain moist, stuff in the gutta-percha forcibly between the teeth, smooth, and let alone to watch every three or six months. Where the cavities are large when you first see them, remove no decay over the pulp. Break down all superfluous walls, saturate with carbolic acid or creosote, force in a lump of the gutta-percha by filling all space as one filling, and let it go until the teeth have become so far separated by the act of mastication—not by expansion of the material—as to have replaced with another or a patch on the surface. Now, here is the point I wish to make that you have never recognized as a factor, because you have ignored the laws of articulation.

By this means I save from future decay and the risk of pulp exposure; but, above all else, I give a condition that enables the child to use with impunity every part of the jaws with hard or soft food, and no pain or fear of it, which no other plan could offer. And, above all this, I drive the first permanent molar so much further back upon the ramus that, the nearer it is to the condyle or point of motion, the wider it keeps the jaws apart at the incisors, and prevents absolutely the too great encroachment of the lower upon the palatal surfaces of the superior incisors, which, if allowed, would destroy normal articulation,—make too deep an overbite and underbite, and, withal, cause an overlapping of the inferior incisors and the full use of the jaw teeth, because, in the lateral movements of the lower jaw, the incisors would strike first too long before the molars could come in contact, and really only the up-and-down movement would be attained.

### Must Grasp Articulation.

This you could never know, nor can you appreciate now, unless you fully grasp the laws of articulation. This has never been taught, and, save a few followers of my special friends, is not practised.

This was a revelation to me when, in 1858, the articulator was born. And, as soon as the pink gutta-percha made its appearance, with rubber plates for trial or base plates, before they were brought forth, I struck upon this treatment and have followed it ever since; and the results have proved I have but few cases of irregularity in my own immediate practice, and those but simple ones, and seldom a pulp exposed for treatment, and but few demoralized subjects, and a brighter future for the permanent set, with plenty of room and to spare for them to come in. Should decay occur on the anterior approximal surface of the first permanent molar, I prevent its spread and in many cases anticipate or treat it superficially; and, if to fill, do so when I have all the results I want,—but seldom with gold even then, as I do not know the exact position the second bicuspid will take; besides, most of the cavities are very small and not susceptible of contouring over all the approximal surface, which has to be done if we contour at all on any surface. From the temporary incisors of children I generally remove caries when small, and do not scruple to fill with amalgam if they need filling. I have never used the dam for any child except when the permanent molars required filling.

Lastly, to detect the incoming permanent tooth when the temporary one shows no sign of its approach, I, at the proper time, use an exploring needle under, or in some cases directly through, the gum to feel for it. It is the precursor of events, and saves much irregularity and fear. Without this precaution many temporary molars that become fastened between the permanent molars and first permanent bicuspids would remain in for years too long. So much for the treatment to the twelfth year. Gutta-percha is my sheet-anchor.

In the permanent incisors anticipation is generally adopted; if decayed, or oxyphosphate or pink gutta-percha is used, never gold. I seldom have any fillings at all in these teeth. The sixth-year molars on buccal surfaces are generally smoothed and decay arrested, or, if to be filled, pink gutta-percha. It is impossible to save every tooth without filling; yet, even with the worst of these cases, *superficial* decay can be arrested and thousands of fillings saved, and our art made a comfort and a blessing.

### Recurrence of Decay at the Cervix.

You all admit that recurrence of decay at the cervical border gives you the greatest difficulty to surmount, and as yet you have not reached the cause nor the remedy. It must be admitted that, if this one thing alone can be mastered, we have overcome our most powerful foe.

It is a fact not to be denied that every dentist cries out for some method to prevent recurrence at the cervix. This is positive proof that everyone has hands full of proximal cavities from the cuspid back. Everyone must admit that contour fillings alone have been the only help or partial cure, although it has to be repeated or requires patching.

A case presents where caries have run wild. Not an approximal surface scarcely but is involved. No pulps quite exposed, but threatening. Every tooth has been filled and refilled, and by more than one dentist. Contour has been attempted. Where the fillings of gold remain they are so undermined there is nothing but utter annihilation unless all are removed. The teeth from their loss of proximate surfaces are all out of articulation, which can be best seen by taking an impression and putting the casts in my articulator. Look closely at the cervix, and you will find the root of each so close that no thread can be forced through, and the decay is far up under the cervix. Look further and probe for the alveolar process, and not a vestige of it remains for a quarter of an inch up. Look also to the second molar where the first has been extracted, and on that side the process is gone far down and nothing but loose gum tissue remains, and is constantly receding, and whenever a tooth has been lost the process about the cervix absorbs as the body of the jaw absorbs.

In this state of affairs you put on your dam and separator, and you obtain a slight widening and at once fill permanently the excavated tooth. No attention is paid to the articulation of the teeth. You have no desire to wait, and you rush on headlong to fill and get your pay. The rest of the teeth are left without anything in them until one by one you have had you your patient at least twice a week for months, two hours or more at a sitting, until they are exhausted and condemn dentistry, and while you are rushing through to complete every cavity with a filling you have done nothing to prevent further rapid decay, and pulps become exposed and the patient has to suffer.

Now, I know this is the case with nearly every man's practice. I see it every week, and I know from personal contact in conversations with patients of others who have not come to me for treatment.

Now, you can do better than this and not only retain your patients, but bridge over time as well as space and fill and treat at your leisure.

I will take the same mouth just illustrated, and without placing in one single permanent filling of any kind of metal treat it with pink gutta-percha alone, with a little of the white as a facing, where necessary. I cut out only partially the cavities on one side of the jaw or jaws, always exposing every grinding surface where the approximal is gone and make compound by running all of the cavities into one, seldom leaving any approximal cavity to stand alone, but opening it into the grinding surfaces. This is a cardinal principle with me. There is one surface or border I complete at once, and that is the cervical, so that I never have to touch it again, and this I cut so far up as to not only remove all caries, but where I know the gum and process will grow up and over it. This is finished, and to enable me to do so I forgot to say I never put on the rubber dam in any case until I fill permanently, when the cervix is firm and will admit of its adjustment. It is easy to stop the blood with perchloride of iron, creosote, or any styptic.

### TREATMENT.

And now for the further treatment. Into all the spaces I have made on one or more sides in one or more teeth I place great pieces of pink gutta-percha, and, with no separation between them in any case stuff the whole intervening space, trim and let alone. This I do until every place is filled in. I dismiss the patient, and have him call in three or six months or a year, as I may please; and, as I find the teeth wide enough apart for a plus-contour filling, and the alveolar gum border and process is in perfect health, and the process has grown up to the gutta-percha, then I fill only those that show that they are far enough apart at the cervix to permit a healthy, full process to grow in order that the gum will have proper substance, and cleave to the root and cover up and over the margin of the filling at the cervix. In this, I tell you, is your future security at the alveolar border.

No one has ever called attention to the difference in width of the approximal spaces at the cervix for the bicuspids and molars. The gutta-percha should remain in until double the width or space is gained between the molars than the bicuspids, on account of the greater size of the molars, where more proximal surface is in contact and no room left for cleansing, unless the spaces are very much greater than normal, and the contour made to suit this issue. Here is where you will say, "You will destroy the articulation and cause greater strain on the fillings and also the teeth." No, you are mistaken. When the whole of these proximal surfaces are filled with the semi-elastic stopping, and the act of mastication set up, the teeth that at first are out of the normal position and only touch on part of their crown surfaces are now allowed to readjust themselves; as the gutta-percha will give where the greatest pressure is brought to bear, and where least resistance is offered, no change occurs. I am not mistaken in this. Try it, and watch a few cases if you cannot believe me.

### COMPENSATION.

This method is a test for any further treatment, which, if needed, can so easily be done. It permits of weeks, months and years before the permanent filling need be introduced. No danger of decay, none of loss of structure from fracture. And, in fact, you can dismiss the patients thus treated with the greatest indifference as to the issue. Do you ask whether I charge for all this work, and when I send in my bill? I charge for even my thoughts as well as my work. My patients never object, but often beg me to leave in the gutta-percha.

Thus I practice with all; and I am happy in this, knowing that I do far more good, am not troubled about immediate root filling,—fillings falling out,—"conservative treatment of dental pulp." Nor does pyorrhœa ever invade upon my domain of original work, because I know the value of articulation, and how to make every tooth perform its individual and collective function, and no undue pressure given it to press or work its life out of it and give rise to the denudation of the peridental membrane; nor is the food ever found pressing up into the cervical border and remaining, nor the cervix so weakened by want of contact with firm alveolar processes, and the gum is left to hug the root at this vital portion so tightly that nothing ever creeps in to cause recurrence at the cervix.

### SHOULD BE SUED.

Any dentist who allows his original patient who follows orders to have pyorrhœa should be sued for damages. See that no food presses on the gum border; see that no tooth is unduly pressed and contorted by false articulation, caused by improper width and contouring; allow no biting of threads, cracking of nuts, biting of ice upon one tooth only; or, when a tooth, or teeth, has been lost, see that the articulation is restored, and my word for it gout or no gout, syphilis or disease, pyorrhœa will not come, except filth and malaise of one or more teeth.

Gutta-percha used as matrices for gold, amalgam, or oxyphosphate fillings I will not dwell upon; you need nothing better. For holding teeth in position after correction where there are cavities in both, I need only mention it. As for assistance on the temporary and permanent teeth, to keep the ligature from slipping down on the cervix by carrying the ligature through it; for fastening pins into roots for crowns; as a medium between crowns and roots to prevent further caries; as a protection to all roots when a gold crown is used; and, in fact, as a factor in our practice, there is nothing to fill its place.

In only one instance it will not do. Never place it in contact with an amalgam filling, especially when it is covered up over a nearly exposed pulp, for it will oxidize the amalgam and discolor it and make it valueless at the margin. White gutta-percha is not so. The sulphur in the pink will do the work; hence, I say, this contact with amalgam is a failure. I am in love with it, and without it I would be lost.—*International.*

---

ARTICLE II.

